# Barriers to Effective Clinical Experiences Among Newly Qualified Registered Nurses: A Descriptive Qualitative Study

**DOI:** 10.3390/healthcare13182343

**Published:** 2025-09-17

**Authors:** Meluleki Zondi, Sipho Wellington Mkhize

**Affiliations:** Discipline of Nursing, School of Nursing and Public Health, University of KwaZulu-Natal, Durban 4041, South Africa; mkhizes4@ukzn.ac.za

**Keywords:** newly qualified nurses, transition to practice, transition shock, mentorship, rural healthcare

## Abstract

**Background:** The transition from student to professional nurse is often overwhelming for newly qualified registered nurses, especially in rural and resource-limited settings. Systemic barriers such as staff shortages, limited resources, and lack of mentorship hinder their ability to gain effective clinical experiences. This gap threatens both the professional development of newly qualified registered nurses and the quality of patient care, justifying the need for this study. **Aim:** This study aimed to explore how the shortage of resources and functional infrastructure affects the clinical experiences of newly qualified registered nurses. **Methods:** A descriptive qualitative design was employed, grounded in an interpretivist paradigm. Data were collected through three semi-structured focus group interviews with a purposive sample of 25 NQRNs. A rigorous thematic analysis, following the Braun and Clarke framework, was used to identify, analyze, and report patterns within the data. **Results:** The analysis revealed a complex interplay of six interconnected themes that define the NQRNs’ experiences: (1) an institutional void of clinical support and mentorship; (2) systemic failures in management and leadership; (3) crippling resource constraints and infrastructure decay; (4) pervasive emotional and psychological distress; (5) a trajectory towards professional burnout; and (6) profound job dissatisfaction and disillusionment. These barriers were found to collectively undermine clinical confidence, compromise patient safety, and threaten nurse retention. **Conclusions:** NQRNs in the Chris Hani District are navigating a “perfect storm” of systemic failures that hinder their professional development and personal well-being. The findings highlight an urgent need for multi-level interventions, including the implementation of standardized mentorship programs, leadership development for nurse managers, strategic investment in rural health infrastructure, and the establishment of formal mental health support systems. Addressing these foundational issues is paramount to building a resilient nursing workforce and ensuring equitable healthcare delivery.

## 1. Introduction

The transition from the structured, protected environment of academic nursing education to the dynamic and often chaotic reality of professional practice is a critical and universally challenging period for newly qualified registered nurses (NQRNs) [1,2]. This phase, frequently described as a “reality shock” or “transition shock,” is characterized by a significant gap between theoretical knowledge and the practical demands of clinical care [3,4]. Globally, healthcare systems grapple with supporting NQRNs as they navigate immense professional, intellectual, and emotional adjustments. The World Health Organization’s “State of the World’s Nursing 2020” report underscores the importance of positive practice environments and effective support for early-career nurses to ensure workforce stability and quality of care [5]. Failure to adequately support NQRNs during this vulnerable period contributes to high rate of burnout, job dissatisfaction, and attrition, with some studies indicating that up to one-third of nurses leave their first position within two years [6].

A seminal theoretical framework for understanding this phenomenon is Duchscher’s Theory of Transition Shock [4]. This theory posits that NQRNs undergo a tumultuous process involving distinct phases of loss, doubt, confusion, and disorientation as they reconcile their idealized expectations with the complex realities of the workplace. The theory highlights the need for structured support systems, such as preceptorships and mentorships, to help NQRNs move from a state of shock to one of growth and professional integration. Without such support, NQRNs are at high risk of emotional exhaustion and feeling professionally incompetent, which directly impacts patient safety and care outcomes [7].

In South Africa, these global challenges are magnified by a unique and complex socio-economic and political landscape. The nation’s healthcare system is a dualistic entity, characterized by a well-resourced but exclusive private sector and an overburdened, under-resourced public sector that serves over 80% of the population [8]. This disparity is a legacy of the apartheid era and continues to drive significant health inequities, particularly along rural–urban and socio-economic lines [9].

NQRNs in South Africa, who are often required to complete a mandatory year of community service in public facilities, are frequently deployed to the front lines of this strained system [10]. They are placed in primary healthcare clinics and district hospitals in rural and underserved areas, where the burden of disease—including high rates of HIV, tuberculosis, and non-communicable diseases—is immense [11]. These facilities are often plagued by chronic staff shortages, a lack of essential medical equipment and supplies, and dilapidated infrastructure [7,12]. Consequently, NQRNs are not merely transitioning into practice; they are transitioning into a system in crisis.

The Chris Hani District in the Eastern Cape Province epitomizes these challenges. It is one of South Africa’s most impoverished districts, with high unemployment and a heavy reliance on public health services [13]. The healthcare facilities in this region face a constant struggle to provide adequate care amidst severe resource limitations. For an NQRN, starting a career in such an environment presents a formidable test of resilience, skill, and professional identity. They are expected to provide safe, quality care while navigating systemic failures that are far beyond their control.

While a growing body of literature explores the transition experiences of NQRNs, much of it has been conducted in well-resourced settings in high-income countries [14]. These studies consistently describe the challenges of “transition shock,” including role adaptation, professional identity formation, and clinical competence development. However, there remains a critical gap in in-depth, contextualized research that examines the lived experiences of NQRNs in profoundly resource-limited, rural settings within sub-Saharan Africa, where systemic challenges such as staff shortages, high patient acuity, and limited mentorship exacerbate the difficulties of transition [15,16].

This study seeks to address this gap by exploring the specific barriers affecting the clinical experiences of NQRNs in the Chris Hani District, a rural district in South Africa. The aim is not only to describe the challenges but also to provide a nuanced understanding of how these barriers intersect and collectively shape the professional journey of new nurses. By giving voice to these NQRNs, this research intends to generate evidence to inform the development of targeted, context-appropriate support strategies and policy recommendations aimed at strengthening the nursing workforce and improving healthcare delivery in South Africa’s most vulnerable communities.

### 1.1. Objective

The objective of this study was to explore the barriers that hinder effective clinical experiences among newly qualified registered nurses in the Chris Hani District of the Eastern Cape, South Africa.

### 1.2. Research Question

What are the barriers that newly qualified registered nurses encounter in achieving effective clinical experiences within resource-limited, rural healthcare settings?

## 2. Materials and Methods

This study employed a descriptive exploratory and contextual qualitative research design, as it focused on discovering and gaining insights about NQRNs experiences towards the transitioning stage. The participants described their challenges about the limited resources, shortage of staff, and lack of mentorship. Focus group interviews were conducted in the naturalistic setting to attain in-depth information on their challenges as NQRNs.

### 2.1. Study Setting

This study was conducted across two public hospitals located within the Chris Hani District, Eastern Cape Province, South Africa. This district is geographically vast and predominantly rural, with a population of approximately 828,000 people, who face significant socio-economic challenges [13]. The public hospitals in this district (Komani Hospital and Frontier Hospital being the primary recruitment sites) serve as the main providers of healthcare for the majority of the population. High patient loads, severe shortages of healthcare professionals, chronic underfunding, and aging infrastructure, characterize these facilities. The Chris Hani District, in particular, faces persistent staff shortages, with nurse-to-patient ratios far below the national average (Section27, 2024). This context provided a highly relevant setting to explore the lived experiences of NQRNs working under conditions of extreme resource scarcity. Facilities in the district frequently experience inadequate supplies of essential medication and equipment [17], alongside vacancy rates for professional nurses that remain disproportionately high in rural Eastern Cape health services [18].

### 2.2. Participants and Sampling Strategy

A purposive sampling strategy was utilized to recruit participants who could provide rich and relevant information pertinent to the research question [19]. The inclusion criteria were (1) being a registered nurse; (2) having between 1 and 5 years of post-qualification clinical experience; (3) NQRNs not employed within the Department of Health under the Chris Hani District. The exclusion criteria were (1) operational managers; (2) registered nurses who have six years of experience and above; (3) NQRNs who do not work at the Department of Health under the Chris Hani District. This study included 25 participants, and data collection continued until saturation was reached, which occurred at the second focus group (participant 13); a third group was conducted to confirm findings and ensure a breadth of perspectives. Semi-structured interviews lasted between 40 and 60 min, and all participants provided consent for audio recording. The interviews were transcribed verbatim by the researcher, and the transcripts were reviewed against the recordings for completeness and anonymized prior to analysis. To protect confidentiality, participants’ names and identifying details were removed, and each was assigned a code number. The researcher adhered to the Protection of Personal Information Act 4 of 2013. The audio files and transcripts were stored securely on a password-protected computer accessible only to the research team. Data will be retained for five years and then permanently deleted. Field notes taken during and immediately after the interviews were incorporated into the data analysis to capture non-verbal cues, contextual factors, and researcher reflections, thereby enriching the interpretation and supporting the triangulation of emerging themes.

### 2.3. Data Collection

Data were collected through three in-depth, semi-structured focus group interviews conducted on February 2025. Focus groups were chosen as the primary data collection method to facilitate a dynamic discussion where participants could build on each other’s ideas, share common experiences, and explore different perspectives [20]. Each focus group consisted of six to eight participants and lasted between 40 and 60 min.

The primary researcher, who used a semi-structured interview guide, facilitated the interviews. The guide included open-ended questions designed to explore key areas identified in the literature as central to the transition experiences of newly qualified registered nurses. These areas included orientation and induction, mentorship and management support [21], resource availability and workload [22], and emotional well-being and job satisfaction [23]. These domains were chosen because they have been shown to significantly influence professional adjustment, clinical competence, and retention, particularly in resource-constrained and rural healthcare settings. Example questions included “Can you describe your experience of being mentored or supervised when you first started working here?” and “How have resource shortages, if any, affected your ability to provide care?”. Probing questions were used to encourage participants to elaborate on their responses. All interviews were audio-recorded with participants’ explicit permission and were conducted in private meeting rooms at the hospitals to ensure confidentiality. Field notes were also taken by the researcher to capture non-verbal cues and contextual details.

### 2.4. Data Analysis

During the analysis of the two focus group sessions, the coding process revealed a high degree of repetition across participants’ responses. By the end of the second session, no new codes or themes were emerging; instead, the discussion reinforced and deepened the themes already identified in the first session. The coding framework had stabilized, and further sessions were unlikely to yield substantially different insights. Therefore, saturation was deemed to have been achieved after the second focus group. The researcher transcribed the data verbatim. Field notes provided rich descriptions and contextual details that can be coded and analysed alongside the interview transcripts. A rigorous thematic analysis was conducted following the six-phase framework outlined by Braun and Clarke [24]. This inductive approach allowed themes to emerge directly from the data rather than being imposed by pre-existing theories.

- Phase 1: Familiarization: The researchers immersed themselves in the data by transcribing the audio recordings verbatim, reading and re-reading the transcripts, and listening to the recordings multiple times.
- Phase 2: Generating Initial Codes: The transcripts were systematically coded line-by-line. Initial codes were generated to capture interesting features of the data relevant to the research question (e.g., “no one to ask,” “broken BP machine,” “feeling scared”).
- Phase 3: Searching for Themes: The codes were collated and sorted into potential overarching themes. The researchers looked for broader patterns of meaning and relationships between the codes.
- Phase 4: Reviewing Themes: The potential themes were reviewed and refined. This involved checking the themes against the coded data extracts and the entire dataset to ensure they accurately represented the participants’ experiences.
- Phase 5: Defining and Naming Themes: Once refined, each theme was clearly defined, and a concise, descriptive name was assigned. The essence of what each theme was about was articulated.
- Phase 6: Producing the Report: The final phase involved writing the narrative analysis and weaving together the analytic narrative with compelling participant quotes to illustrate the themes. The analysis was conducted collaboratively by the research team to enhance rigor.

### 2.5. Ethical Considerations and Trustworthiness

This study received ethical approval from the University of KwaZulu-Natal Humanities and Social Sciences Research Ethics Committee (HSSREC/00007754/2024) and the Eastern Cape Health Research Committee (EC_202410_033). Written informed consent was obtained from every participant after they received a full explanation of the study’s purpose and procedures and their rights. To ensure trustworthiness, this study followed the criteria outlined by Nowell [25]. Credibility was enhanced through prolonged engagement with participants and peer debriefing sessions among the researchers. Dependability was maintained via a detailed audit trail documenting all research activities. Confirmability was strengthened through the use of direct participant quotes and a reflexivity journal maintained by the primary researcher. Transferability was addressed by providing a thick description of the context and findings, enabling readers to assess the applicability of the results to other settings.

## 3. Results

The NQRN participants had diverse educational backgrounds and clinical experiences. Specifically, thirteen NQRNs held diplomas in nursing, and twelve NQRNs had earned bachelor’s degrees in nursing. The age of the participants ranged from 24 to 40 years, and their clinical practice experience as NQRNs spanned from 1 to 5 years (refer to Table 1 for detailed demographics).

The thematic analysis of the focus group interviews revealed six major themes that encapsulate the profound and interconnected barriers faced by NQRNs in the Chris Hani District. Table 2: The research own creation design.

### 3.1. Theme 1: An Institutional Void of Clinical Support and Mentorship

A universal and deeply felt sentiment among participants was the profound lack of structured clinical support and meaningful mentorship. Rather than a supported transition, they described an abrupt and isolating immersion into highly demanding roles, a phenomenon they repeatedly referred to as being “thrown in the deep end.” This void of guidance was particularly acute during their initial months of practice, leaving them feeling professionally vulnerable and unprepared.

#### Subtheme: Inadequate Orientation and Supervision

Participants reported minimal or no structured orientation when starting their roles, leaving them uncertain about expectations and workflows. Supervision by senior staff was often inconsistent or absent, forcing new nurses to navigate complex clinical situations alone. This lack of guidance contributed to anxiety, reduced confidence, and frequent reliance on self-directed learning:

*“There was no orientation. On my first day, they just showed me the ward and said, ‘This is your ward, these are your patients.’ I was terrified. I had no idea who to ask if I had a problem, because everyone else was just as busy and stressed.”*

*(P1)*

*“Mentorship is a nice word we read about in textbooks. Here, it doesn’t exist. The senior nurses are either burnt out or they see you as a threat. You learn by making mistakes, and you pray those mistakes don’t harm a patient. It’s a very hard way to learn.”*

*(P3)*

*“I remember a time I needed to do a procedure I had only seen once in college. I asked a senior sister for help, and she told me, ‘You are a professional nurse now, figure it out.’ I ended up having to Google it on my phone in the sluice room, feeling like a complete failure.”*

*(P2)*

### 3.2. Theme 2: Systemic Failures in Management and Leadership

Participants described a significant disconnect between themselves and the hospital management. This was characterized by a lack of visibility, poor communication, and a perceived indifference to the daily struggles of frontline staff. Management was often seen as a bureaucratic entity that imposed policies without understanding the clinical realities, further exacerbating feelings of frustration and disempowerment.

#### Subtheme: Inadequate Training and Skill Development

Training opportunities were described as inconsistent and poorly implemented, limiting professional growth:

*“We had a training session on a new electronic system. They sent one manager, who then was supposed to train all of us. The training never happened properly. It’s always like that. Opportunities for skills development are there, but they don’t reach the people who actually need them on the ground.”*

*(P7)*

*“Even when workshops are scheduled, they often conflict with our shifts. We miss out because management doesn’t coordinate with staff availability.”*

*(P11)*

*“When there’s a critical incident, like a patient fall or a medication error, management’s first reaction is to find someone to blame. There is no culture of supportive, non-punitive incident reporting. It makes you afraid to speak up, so problems just get hidden until they become disasters.”*

*(P5)*

### 3.3. Theme 3: Crippling Resource Constraints and Infrastructure Decay

This theme was one of the most dominant and emotionally charged in the discussions. Participants detailed a daily battle against a severe and chronic shortage of basic and essential resources, ranging from medical equipment and supplies to functional infrastructure and adequate staffing. This constant scarcity not only compromised their ability to provide safe and effective care but also eroded their professional morale.

#### Subtheme: Shortage of Equipment and Staff

Participants described a pervasive shortage of essential medical equipment and insufficient staffing as a major barrier to providing safe and effective patient care. Limited access to functioning equipment, such as vital sign machines, often forced nurses to wait or improvize, compromising patient monitoring and treatment. Chronic understaffing, particularly during night shifts, meant that single nurses were responsible for entire wards, performing admissions, drug rounds, emergencies, and paperwork simultaneously. These conditions not only endangered patients but also contributed to professional stress and low morale:

*“We have one working vital signs machine for a ward of 40-plus patients. You spend half your shift just waiting for the machine. How can you monitor a critically ill patient properly like that? It’s impossible. We are set up to fail.”*

*(P6)*

*“The staffing is a nightmare. It’s normal to be the only registered nurse for the entire ward at night, with one nursing assistant. You have to do everything admissions, drug rounds, emergencies, paperwork. The patient-to-nurse ratio is not just unsafe; it’s inhumane for both the patient and the nurse.”*

*(P2)*

*“Forget advanced equipment; sometimes we don’t have the basics. There are days we run out of sterile gloves, or we don’t have enough linen. The roof in our ward leaks when it rains. We are working in conditions that feel like they are from another century.”*

*(P3)*

*“The shortage of staff is really putting a lot of pressure on us, while the workload is too hard it is really hard to work here.”*

*(P8)*

### 3.4. Theme 4: Pervasive Emotional and Psychological Distress

The cumulative effect of the preceding barriers manifested as profound emotional and psychological distress. Participants spoke of experiencing constant fear, anxiety, and a sense of hyper-vigilance. The fear of making a fatal error in such a high-stakes, low-support environment was a heavy burden, impacting their mental health both at work and in their personal lives.

#### 3.4.1. Subtheme: Fear and Anxiety

Participants reported pervasive fear and anxiety stemming from the high-stakes nature of their work and the lack of adequate support. The constant pressure to manage critically ill patients alone, often with limited equipment and staffing, created a state of hyper-vigilance that affected their mental well-being both at work and at home:

*“I have anxiety every single day before I come to work. My stomach is in knots because I’m so scared of what I might face a patient crashing and I’m alone, or a piece of equipment failing during an emergency. It’s a constant state of fear.”*

*(P1)*

*“It affects your personal life. You go home exhausted, not just physically but emotionally. You are irritable with your family. You can’t sleep because you are replaying everything that happened on your shift, thinking about what you could have done differently if only you had more time or more help.”*

*(P2)*

#### 3.4.2. Subtheme: Inadequate Leadership and Emotional Support

The lack of accessible leadership and structured emotional support exacerbated participants’ distress. Without debriefing sessions, counseling, or guidance from senior staff, NQRNs often felt isolated in coping with the emotional toll of trauma, patient deaths, and high-pressure clinical responsibilities:

*“The emotional toll is immense. I’ve seen so much trauma and there’s no one to talk to about it. There’s no debriefing, no counseling. You are just expected to be strong and carry on. I have cried in my car after a shift more times than I can count.”*

*(P6)*

*“They is a huge gap that the managers need to do, to guide and support us, we are dying with stress and workload while they is no support are we getting.”*

*(P13)*

*“ They understand that we are still knew but they is no support that we get from the supervisors, they will rush to blame when the mistake has occurred. That also put much pressure to us as new nurses.”*

*(P9)*

### 3.5. Theme 5: A Trajectory Towards Professional Burnout

Participants described symptoms and experiences synonymous with professional burnout—emotional exhaustion, depersonalization, and a diminished sense of personal accomplishment. They felt that the relentless workload, coupled with the lack of support and resources, was pushing them beyond their limits, leading to absenteeism and a desire to leave the profession or, at the very least, the rural public sector.

#### Subtheme: High Workload Stress

Participants highlighted that relentless workloads and staffing shortages were major contributors to their professional burnout. Managing entire wards alone, performing multiple clinical and administrative tasks, and coping with inadequate resources created chronic stress, emotional exhaustion, and a sense of depersonalization. These conditions not only threatened patient care but also led many NQRNs to consider leaving the profession or the rural public healthcare sector:

*“I am burnt out. Completely. Some days I feel like a robot, just going through the motions. I don’t feel the same empathy I used to. It’s a defense mechanism, I think. If you feel too much, you won’t survive.”*

*(P6)*

*“Burnout is why people are always on sick leave. People aren’t faking it; they are mentally and physically broken. The system runs you into the ground and then wonders why there’s a staffing crisis.”*

*(P3)*

*“I am actively looking for a way out. Maybe go overseas, or work for a private hospital, or just leave nursing altogether. I love being a nurse, but I can’t sacrifice my own health and sanity for a system that doesn’t care about me.”*

*(P7)*

### 3.6. Theme 6: Profound Job Dissatisfaction and Disillusionment

The final theme captures the deep sense of disillusionment that permeated the participants’ narratives. There was a stark and painful contrast between the standard of nursing care they were taught to provide and the compromised care they were forced to deliver. This gap between their professional ideals and the harsh reality of their practice led to widespread job dissatisfaction and a loss of pride in their work.

#### Subtheme: Poor Job Satisfaction

Participants expressed profound dissatisfaction with their work, stemming from the gap between the high standard of nursing care they were trained to provide and the compromised care they were able to deliver in practice. Task-oriented, crisis-driven work environments, combined with systemic constraints, left them feeling ineffective, undervalued, and disillusioned. This mismatch between professional ideals and workplace realities significantly eroded their pride, motivation, and overall job satisfaction:

*“I am not proud of the nursing care I give most days. I know it’s not my fault, but it’s my name on the patient’s chart. We were trained to be advocates for our patients, to give holistic, high-quality care. What we do here is just task-based crisis management.”*

*(P1)*

*“The biggest challenge is feeling like you are not making a difference. You are just plugging holes in a sinking ship. You go home feeling defeated. That feeling of dissatisfaction is what kills your spirit.”*

*(P2)*

*“Is this what I studied so hard for? To work in these conditions? I feel cheated. I feel like the system has failed me, and in turn, it is failing the patients who depend on us. It’s a deep, deep dissatisfaction.”*

*(P5)*

The findings highlight how inadequate support, systemic failures, resource shortages, emotional strain, and professional burnout collectively shape the early experiences of newly qualified registered nurses. These insights underscore key barriers to effective practice and areas requiring targeted interventions. The next chapter discusses these findings in relation to the existing literature and considers their implications for nursing practice, education, and workforce planning in resource-constrained settings.

## 4. Discussion

This study provides a granular and sobering account of the barriers confronting NQRNs in the Chris Hani District, painting a picture of a system under severe strain where new professionals are set up for failure. The six themes identified are not isolated issues but are deeply interconnected, creating a “perfect storm” that profoundly impacts NQRNs’ professional development, psychological well-being, and retention. This discussion elaborates on these findings by integrating the recent literature, focusing on the individual and psychological toll of these systemic failures, and providing a more profound analysis of the implications.

### 4.1. The Failure of Transition Support: From Theory to a Harsh Reality

The overwhelming sentiment of being “thrown in the deep end” directly reflects the concept of “transition shock” [26]. The absence of structured orientation, preceptorship, and mentorship programs denies NQRNs the critical scaffolding needed to bridge the gap between theory and practice. The literature consistently demonstrates that effective mentorship is a key predictor of NQRN competence, confidence, and job satisfaction [27]. The findings of this study suggest a complete breakdown of this support mechanism. This is not necessarily due to a lack of willingness from senior nurses but is often a symptom of a burnt-out, over-stretched workforce, where experienced staff lack the time, energy, or formal training to mentor effectively [28]. This creates a vicious cycle: unsupported NQRNs struggle, which increases the burden on senior nurses, further diminishing their capacity to provide support. This lack of guidance fosters a constant state of fear and anxiety, depleting the new nurses’ psychological resources and making them susceptible to burnout [29].

### 4.2. Ineffective Leadership as a Catalyst for Systemic Dysfunction

The perceived detachment and lack of support from management aligns with research on the critical role of resonant leadership in creating positive practice environments [30]. When leadership is seen as punitive and unresponsive, it fosters a culture of fear and silence, which is antithetical to patient safety and quality improvement [31]. The failure to cascade training opportunities and to engage with frontline staff on clinical challenges indicates a significant leadership deficit. In a high-pressure environment, visible, supportive, and authentic leadership is not a luxury but a necessity for maintaining staff morale and fostering a culture of resilience and continuous learning. The absence of such leadership in the participants’ experiences served as a major catalyst for their feelings of disempowerment and frustration, directly impacting their intention to remain in their positions [31,32].

### 4.3. Resource Scarcity and the Individual Nurse: A Source of Moral Injury

The crippling resource constraints described by participants are emblematic of the deep-seated inequities within the South African healthcare system [9,33]. Working in an environment devoid of essential tools and adequate staff forces nurses to practice “compromised care”—a form of practice where they are knowingly unable to meet professional standards due to systemic limitations [12,34]. This has devastating consequences. It not only directly endangers patients, but it also inflicts a moral injury on nurses, who are caught between their professional ethics and the constraints of their environment [34]. The individual nurse is left to grapple with feelings of guilt, shame, and a diminished sense of professional worth. This internal conflict is a significant contributor to the emotional and psychological distress reported by participants. The chronic nature of these shortages in the Chris Hani District suggests a systemic failure in public health funding, supply chain management, and human resource planning at the provincial and national levels [5,35].

### 4.4. The Inevitable Human Cost: Distress, Burnout, and the Erosion of Professional Identity

The pervasive emotional distress and trajectory towards burnout are the inevitable human consequences of these systemic failures [29]. The combination of a high workload, low control, and inadequate support is a classic recipe for burnout [31]. What this study adds is a stark illustration of how these pressures are intensified in a resource-deprived, rural context, leading to a profound impact on the individual’s professional identity [36]. New nurses enter the profession with a set of ideals and a desire to provide compassionate, high-quality care. When the reality of their practice is one of constant compromise and crisis management, this idealized professional identity is violated [36]. This leads to disillusionment and a loss of pride in their work, as articulated by the participants. The desire to leave the profession or the public sector, expressed by many participants, highlights a critical threat to the sustainability of the rural nursing workforce [35,37]. Each NQRN who leaves represents not only a personal tragedy but also a significant loss to a community that desperately needs their skills [30,31].

### 4.5. The “Perfect Storm”: The Intersectionality of Systemic Barriers and the Role of Individual Resilience

A crucial insight from this study is that these barriers do not operate in isolation; they are intersectional and mutually reinforcing. A lack of mentorship is made worse by high workloads. High workloads are exacerbated by equipment shortages. The stress from these issues is compounded by a lack of management support. This creates a “perfect storm”—a synergistic vortex of dysfunction that makes it nearly impossible for NQRNs to thrive.

While systemic change is paramount, the role of individual moral resilience and coping mechanisms cannot be overlooked [34,36,38,39]. Moral resilience is the capacity of an individual to sustain or restore their integrity in response to moral adversity [34]. However, expecting individual nurses to be endlessly resilient in the face of systemic collapse is both unrealistic and unjust. The findings suggest that while NQRNs attempt to cope, their strategies are often reactive and focused on short-term survival (e.g., “just getting through the shift”) [38]. Without formal support systems to bolster their resilience and professional identity, they are at high risk of compassion fatigue and burnout [34,36]. This intersectional perspective is vital for developing effective interventions; tackling one issue (e.g., providing mentorship training) without addressing the others (e.g., unsafe staffing ratios and lack of resources) is unlikely to yield sustainable improvements.

### 4.6. Limitations of This Study

While this study provides valuable insights, its limitations must be acknowledged. Firstly, the findings are context-specific to the Chris Hani District and may not be directly generalizable to all rural settings in South Africa or elsewhere, although they may be transferable to contexts with similar socio-economic and healthcare system characteristics. Secondly, the use of focus groups may have led to a degree of social desirability bias, where participants might have been hesitant to share their most negative experiences in a group setting. Thirdly, this study only captures the perspective of NQRNs. Future research would be enriched by a mixed-method approach that includes the perspectives of nurse managers, senior nurses, and policymakers to create a more holistic picture of the challenges and potential solutions. The absence of triangulation with other data sources, such as observations or institutional documents, limits the ability to validate the themes beyond participants’ self-reported accounts. Consequently, the findings should be interpreted as exploratory, with future studies encouraged to incorporate multiple data sources to strengthen credibility.

### 4.7. Implications for Policy, Practice, and Education

The findings of this study have urgent and far-reaching implications for multiple stakeholders:

- For National and Provincial Policy: There is an urgent need for a national policy on NQRN transition to practice that mandates standardized, funded, and protected-time mentorship programs in all public facilities [35]. Furthermore, addressing the rural–urban inequity in healthcare requires deliberate policy action, including targeted funding for rural infrastructure, improved supply chain logistics, and robust incentive packages to attract and retain healthcare professionals in underserved areas [5,33].
- For Hospital Management and Leadership: Hospital and district-level management must move from a bureaucratic to a supportive leadership model [30]. This involves investing in leadership and management training for nurse managers, focusing on skills such as emotional intelligence, communication, and conflict resolution. Creating psychologically safe environments through visible leadership, regular ward rounds, and the implementation of non-punitive, learning-oriented incident reporting systems is critical.
- For Nursing Education: While academic institutions cannot solve systemic healthcare failures, they can better prepare graduates for these realities. Curricula should incorporate more content on health system science, advocacy, and resilience building [34]. Clinical simulations should include scenarios that reflect resource-limited settings, preparing students to make safe and ethical decisions under pressure. Stronger partnerships between universities and healthcare facilities could also help bridge the theory–practice gap and create more supportive learning pathways.
- For Clinical Practice: The establishment of formal peer support groups for NQRNs could provide a vital outlet for sharing experiences and reducing feelings of isolation. Furthermore, access to confidential mental health services and structured debriefing after critical incidents should be standard practice, not an afterthought. These interventions can help bolster individual resilience and support the preservation of professional identity in the face of challenging work environments [34].

## 5. Conclusions

This study illuminates the profound and intersecting challenges that define the clinical experiences of newly qualified registered nurses in a resource-limited, rural district of South Africa. They are navigating a professional landscape characterized by an absence of mentorship, ineffective leadership, crippling resource shortages, and a culture that fosters distress and burnout. This “perfect storm” of systemic deficits not only undermines their ability to provide safe and effective care but also inflicts a significant psychological toll, leading to moral injury and the erosion of professional identity, and threatening their long-term commitment to the nursing profession and to serving vulnerable communities. Addressing these foundational issues is not merely an operational challenge; it is an ethical imperative. Meaningful and sustainable change requires a coordinated, multi-level commitment from policymakers, healthcare leaders, and educational institutions to invest in building a practice environment where new nurses can survive, thrive, and fulfil their potential.

## Figures and Tables

**Table 1:** Demographic profile of participants.

Variable	Frequency
Gender	
Male	10
Female	15
	
Age	
24 to 30	7
30–34	9
35–40	5
40+	4
	
Qualifications	
Bachelor’s Degree	12
Comprehensive Diploma	13
	
Years of experience	
1–2 years	10
3–4 years	8
5 years	7

**Table 2:** Themes and subthemes of the research findings.

Themes	Subthemes
An Institutional Void of Clinical Support and Mentorship	1.1. Inadequate orientation and supervision
2. Systemic Failures in Management and Leadership	2.1. Ineffective in-service training and skill development
3. Crippling Resource Constraints and Infrastructure Decay	3.1. Shortage of equipment and staff
4. Pervasive Emotional and Psychological Distress	4.1.1. Fear and anxiety 4.1.2. Inadequate leadership and emotional support
5. A Trajectory Towards Professional Burnout	5.1. High workload stress
6. Profound Job Dissatisfaction and Disillusionment	6.1. Poor job satisfaction

## Data Availability

The datasets presented in this article are not readily available as they are part of an ongoing study. The data will be made available upon completion of the study. The manuscript is part of a bigger study for a PhD.
